# Discrimination, stalking, sexual harassment and sexual violence at the university – exploring and predicting pattern-based subcategories among students and staff in a German university sample

**DOI:** 10.1186/s12889-025-25864-6

**Published:** 2025-12-08

**Authors:** Marie-Theresa Kaufmann, Noemi Preisendanz, Jörg M. Fegert, Vera Clemens

**Affiliations:** 1 Department of Child and Adolescent Psychiatry/Psychotherapy, University Hospital Ulm, Ulm, Germany; 2 German Center for Mental Health (DZPG), Partner Site Ulm, Ulm, Germany

**Keywords:** Discrimination, Stalking, Sexual harassment, Sexual violence, Safety, University

## Abstract

**Background:**

Discrimination, stalking, sexual harassment and sexual violence (DSHV) remain pervasive problems at universities. However, systemic investigations from German universities are lacking. This study aimed to assess the prevalence of DSHV using an integrative approach with all forms of DSHV to identify pattern-based subcategories with distinct DSHV-profiles and predictive factors for cluster affiliation for students and staff.

**Methods:**

A total of 2,128 participants (1,358 students; 14% response rate; 753 employees; 42% response rate) completed the online survey between April and July in 2023 for the DSHV-investigation. Hierarchical cluster analysis was applied to find pattern-based subcategories (clusters) and multinomial logistic regression models were used to examine factors influencing cluster affiliation.

**Results:**

Three pattern-based clusters emerged for both students and staff, each with significantly distinct DSHV-profiles: There is an unaffected group, a group with limited DSHV-experiences and a group with extensive DSHV-experiences, especially sexual harassment and sexual violence. Being female, perceiving stronger hierarchical structures at workplace or study field, working or studying for a longer time at the university and studying medicine or natural sciences are associated with an increased risk of belonging to the affected clusters.

**Conclusion:**

This study, including a high number of both, data from students and employees, highlights the prevalence and co-occurrence of different forms of DSHV in academic settings and demonstrates that experience of one type of DSHV often correlates with vulnerability to others. Greater attention should be given to female students in the natural sciences. As DSHV is group-specific, measures should include both global and in particular targeted components based on a risk analysis, and might be helpful for students and staff.

**Supplementary Information:**

The online version contains supplementary material available at 10.1186/s12889-025-25864-6.

## Introduction

Universities are essential institutions for education, research and cultural development within a region and are therefore of particular importance to both individuals and regions (see e.g. [1–3]). Universities should ensure equitable access to educational opportunities (Boliver & Powell, 2021) [4]. It is therefore particularly concerning that universities experience high rates of discrimination, stalking, sexual harassment and sexual violence (DSHV) (e.g. [5]). In some academic disciplines, universities can even be the most vulnerable environments for DSHV throughout one´s career: For example, the study period is when female doctors experience the highest levels of harassment in their careers [6] (Table 1).

**Table 1:** Composition of the analysis sample of students (*N* = 1197)

	^1^Participants (students)
	*N* = 1197
Age (***n*** = 1197)	
18–24 years	789 (66%)
25–29 years	312 (26%)
30 years or older	96 (8%)
Gender (***n*** = 1197)	
female	724 (61%)
male	463 (39%)
diverse/others	10 (1%)
Migration (***n*** = 1179)	
no migration in the family history	916 (78%)
migration in the family history	263 (22%)
Parents‘ university degree (***n*** = 821)	
yes	382 (47%)
no	439 (54%)
Study stage (***n*** = 1193)	
bachelor or similar	523 (44%)
master or similiar	580 (49%)
postgraduate	90 (8%)
no response	4 (< 1%)
Field of study (*n* = 1044)	
natural sciences	365 (31%)
medical subjects	483 (40%)
economics	74 (6%)
psychology	122 (10%)
^2^missing	153 (13%)

^1^Percentage in brackets corresponds to the proportion of the analysis sample

^2^Excluded students denied an answer or study several subjects and can not be categorized

Students and staff affected by forms of DSHV often experience various negative health and work productivity outcomes, [5, 7–10]. Employees also face negative consequences for their career opportunities [11]. In particular the frequency of sexual harassment among female students has been associated with a more negative assessment of the campus climate and a lower likelihood of returning to college [12], further undermining women´s opportunities in society. Multiple experiences — in this case, unwanted sexual contact, sexual intercourse, stalking, and intimate partner violence — may be associated with a diminished academic performance among victims [13]. The extent to which DSHV-experiences influence educational opportunities remains uncertain. Cross-sectional analyses indicate that there has been no improvement in terms of rape at universities in recent years and point to alcohol consumption as a risk factor of rape (Koss et al., 2022). This is of particular relevance as DSHV has recently been trivialized as woke issues characterized by increasing oversensitivity and that it is therefore important to take empirical data as a basis. DSHV at universities represent a widespread international issue. However, existing studies focus narrowly on specific forms of discrimination against isolated groups, providing only a fragmented view of the problem (e.g. [14, 15, 16]): Racial/ethnic discrimination was reported by 5–15% of students in a large-scale US-study, depending on their own race/ethnic identity [17]. A recent study at German universities showed a total prevalence rate of 28% for experiences of discrimination [18]. US studies focusing on stalking examined prevalence rates of almost 20% among female students [19, 20]. Data on the situation at German universities in an EU-wide project with 12,663 participating German female students from 16 universities showed similar results of 23% [21]. Sexual harassment is widespread, with recent data showing that 19% of the students reported sexual harassment caused by university staff and 30% by students in a study in the US [5]. Data from the extensive UniSAFE survey, which included 42,186 participants in 15 European countries, revealed that more than half of participating students (57.7%) and staff (73.7%) experienced at least one form of gender-based violence. Sexual harassment was reported by 29.3% of students and 35.3% of staff and sexual violence by 3.5% of participating students and 1.2% of staff [22]. Feltes and colleagues (2012) found that 54.7% of students experienced sexual harassment and 3.3% sexual violence during their college time. In general, prevalence rates for sexual violence vary depending on how the terms are operationalized. A review of completed rape reported prevalence rates ranging from 0.5% to 8.4% between 2000 and 2015 in the US (Fedina, Holmes & Backes, 2018).

Female gender, field of study, hierarchical structures, migration status and duration of university attendance are recognized as potential risk factors for experiencing DSHV: Fedina and colleagues (2020) reported that 20.2% of cisgender women and 11.9% of cisgender men experienced stalking. Women are disproportionately affected by sexual harassment and sexual violence compared to men [5, 7, 23, 24]. Further, the experience of discrimination and harassment are most prevalent in the Faculty of Medicine [18, 25–28, 29]. Female engineering students were also more likely to experience harassment than their counterparts who were not studying science, engineering or medicine [29].

Hierarchical structures are associated with harassment [30, 31]. Doctoral students, in particular, are burdened by this and are, for example, more vulnerable to sexual harassment [22]. Women are more likely to experience gender-based harassment when there is an imbalance in favor of men in the workplace, while this effect does not occur for men in female-dominated settings [32]. The influence of migration status remains poorly understood in some terms and may be correlated with race/ethnicity: A study on international students found that they are at a higher risk of discrimination [33]. Minorities were more often affected by racial/ethnic discrimination [17] and stalking [19]. Some studies suggest that women of white ethnicity are more likely to experience sexual harassment [34], while others suggest that women of color face higher rates of harassment [35, 36]. Wood and colleagues (2021) found differences in the severity according to race/ethnicity [24].

Existing literature recognizes that family educational background is highly relevant to individual educational attainment [37]. Nowadays, women earn more university degrees than men [38]. Despite the potentially valuable effects of family’s educational background, it has not yet been investigated whether it also serves as a protective factor against DSHV. In addition, research suggests that prolonged university attendance may be associated with a higher risk of experiencing sexual harassment [7, 39].

Previous research has primarily focused on specific forms of DSHV and often limited its scope to either students or staff members (e.g. [5, 35]). Studies rarely examine key risk factors, such as perceived hierarchies, family educational background or migration status (e.g. [27, 32]). Additionally, much of the literature focuses on ‘gender-based violence’ or ‘sexual and gender-based violence’ [8, 40, 41]. However, DSHV encompasses not only gender-based violence but also other forms of discrimination, including those based on religion, age, disability or political opinion. This study therefore aims to examine not only gender-based violence, but also all forms of discrimination to which students and staff may be exposed and investigates connections.

An integrative approach that examines all four forms of DSHV among both students and staff is essential. This approach will provide a comprehensive understanding of DSHV, examine co-occurrence patterns of DSHV experiences and identify distinct groups (clusters) based on similar experiences among students and staff. Furthermore, associations between these clusters and known risk factors, including a newly developed operationalization of perceived hierarchy, are planned. This research also presents an opportunity to clarify the complex relationships between DSHV-types and the potential risk factors of ethnicity and migration status. In exploratory analyses we also examined whether family educational background serves as a risk factor for cluster affiliation.

## Methodology

### Study design

The survey was conducted at Ulm University on behalf of the Executive Board of Ulm University over a period of three months from April 24, 2023 to July 25, 2023. All employees and students were informed multiple times via email through the Equal Opportunities Office, the student councils, social media, flyers and posters. The President of Ulm University sent the first invitation email. The survey was approved by the data protection and ethics committee of Ulm University.

The survey was implemented as an online survey using the ‘Evasys’ system – a web-based, decentralized tool for creating, conducting and managing surveys. The survey took approximately 15 min to complete. Participation was anonymous. An English version was available by clicking on a flag icon.

### Questionnaire

The questionnaire (see: [42], appendix) was developed for this study and adapted from the one used in the ‘UniSAFE’ project, a Europe-wide survey on gender-based violence at universities [42]. Additional questions about discrimination and stalking were added. Discrimination included experiences based on social origin, skin color, nationality, gender, sexual orientation, religion, diseases or age. Experiences of stalking included, for example, unwanted contact or ‘lurking’, surveillance, or observation or following. Sexual harassment included intrusive questions about one’s private life, sexually suggestive comments or jokes and unwanted touching, hugging or kissing. Experiences of sexual violence included, for example, being forced to have sexual intercourse. For all types of experiences, respondents could provide further information if the predefined categories did not apply and another form of DSHV was experienced. The participants at first received questions concerning discrimination, following stalking, sexual harassment and then sexual violence. The types of DSHV-experiences (see Tables S1 and S2; supplement) were assessed using the following response categories: *'No*', *'Yes, in the past 12 months*' and *'Yes, in the past 12 months and before*'. and *'I do not want to answer'*. The two affirmative responses were combined into a single ‘*Yes*’ category for analysis. The category *'I do not want to answer'* was assigned to participants who initially agreed to answer the respective section of the questionnaire (e.g. discrimination) but then left the questions in that section unanswered. Participants who refused to answer to at least one of the four areas of experience were excluded. Furthermore, the following sociodemographic categories and study/job description categories were recorded: gender for both samples (female/male/diverse), age for students (18–24 years, 25–29 years, 30 or older), age for staff (18–35 years, 36–50 years, more than 50 years), stage of study for students (bachelor or equivalent, master or equivalent, postgraduate), field of study for students (natural sciences, medical subjects, psychology, economics), duration of employment for staff (< 1 year, 1–5 years, 6–10 years, > 10 years), leadership position for employees (yes/no), parents’ university degree (at least one parent has studied yes/no) and migration in the family history (at least one parent or oneself has migrated to another country yes/no) for both samples.

### Sample

A total of 2128 people participated in the survey. Participants who indicated their gender, age and status (student or employee) and who provided information about the experience or absence of at least one of the target items on DSHV were included in the analyses. 17 individuals did not meet these criteria and were excluded from the analyses. This resulted in the following response rates: Of the 9,477 students at Ulm University, *N* = 1,358 participated. This corresponds to a response rate of 14% for students. Of all 1,783 employees, *N* = 753 participated. This corresponds to a response rate of 43% for employees. Individuals who could be classified as both students and employees (e.g. student assistants) were assigned to the student sample. In total, data were examined for *n* = 1,197 students’ and *n* = 653 employees who responded to all four forms of DSHV. Most of the students were 18–24 years old (66%) and female (61%). 22% reported a family history of migration and 47% had at least one parent who had studied. Most of the participating students were studying a natural science or medical subject (31–40%) (see Table 1). Most of the participating employees were female (60%) and 18–35 years (40%) old. 21% reported a family history of migration and 62% had at least one parent who had studied. Most of the employees worked at Ulm University for more than ten years (39%). A total of 18% had a leadership position (see Table 2).

**Table 2:** Composition of the analysis sample of staff (*N* = 653)

	^**1**^**Participants (staff)**
	*N* = 653
Age (***n*** = 653)	
18–35 years	261 (40%)
36–50 years	200 (31%)
> 50 years	192 (29%)
Gender (***n*** = 650)	
female	394 (60%)
Male	256 (39%)
diverse/others	3 (1%)
Migration (*n *= 638)	
no migration in the family history	502 (79%)
migration in the family history	136 (21%)
Parents’ university degree (***n*** = 635)	
yes	392 (62%)
no	243 (38%)
Duration of employment (*n* = 644)	
< 1 year	62 (10%)
1–5 years	228 (35%)
6–10 years	100 (15%)
> 10 years	254 (39%)
Leadership position (***n*** = 628)	120 (18%)

^1^Percentage in brackets corresponds to the proportion of the analysis sample (*N* = 653)

### Statistical analysis

Data analyses were conducted using the statistical software IBM SPSS Version 21. Analyses of gender issues included only male and female respondents due to the small number of people who selected the category ‘*diverse*’ or ‘*other*’. Furthermore, the response option *‘I do not wish to answer’* was subsequently excluded from further analyses. Results were considered significant at *α* = 0.05. All analyses were conducted separately for students and employees.

### Identification of pattern-based subcategories with different DSHV profiles

To categorize individuals into pattern-based subcategories (clusters), the total number of experiences in each DSHV-category (discrimination, stalking, sexual harassment and sexual violence) was calculated separately and then used as input for an agglomerative hierarchical clustering algorithm (HCA) using the Ward error sum-of-squares agglomeration method with Euclidean distance between individuals. The final cluster solution was derived from the resulting dendrogram. To validate the result, crosstables were computed to test whether the solution of group membership in a pattern-based subcategory was associated with overall frequency per experience (discrimination, stalking, sexual harassment and sexual violence). Chi-square tests for the independence of categorical variables were calculated. Cramér's *V* (for contingency tables of any size) was used to gauge effect sizes, with a value of 0.1 indicating a small effect, 0.3 indicating a medium effect and 0.5 indicating a large effect [43, 44].

### Prediction of cluster affiliation

Multinomial logistic regression models were calculated to predict cluster affiliation. First, chi-square tests were computed for the factors gender, age, migration in the family history, parental university degree, study stage and field of study for the students and gender, age, migration in the family history, parents’ university degree, leadership position and duration of employment for the staff. Kruskal–Wallis tests were calculated for the hierarchy in the field of study (for students) or in the workplace (for employees) – depending on the sample. In a second step, only factors that showed significant relationships in the chi-quare and Kruskal–Wallis tests were used as predictors in the final multinomial logistic regression model. This selection ensures that the regression model includes only predictors with a proven association with cluster affiliation. For the student sample, age was excluded from the model because it was highly correlated (> 0.3) with study stage, which was more relevant to our research question.

## Results

### Prevalence of DSHV

Among students, 40% reported experiences of discrimination, 13% reported experiences of stalking, 33% reported experiences of sexual harassment and 4% reported experiences of sexual violence. Among employees, 46% reported experiences of discrimination,18% reported experiences of stalking, 29% reported experiences of sexual harassment and 6% reported experiences of sexual violence.

### Pattern-based subcategories with different DSHV-profiles among students and staff

**Fig. 1:**
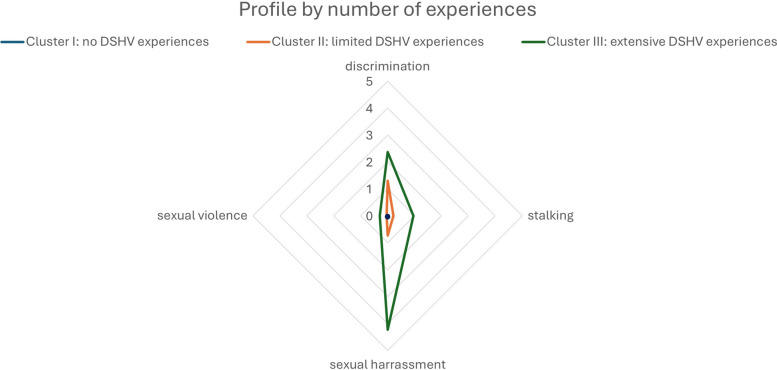
DSHV-profiles by number of experiences among students. Means per cluster were calculated

**Fig. 2:**
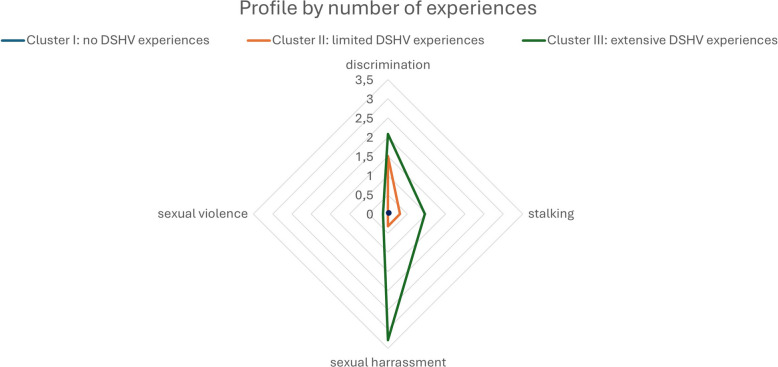
DSHV-profiles by number of experiences among staff. Means per cluster were calculated

A hierarchical cluster analysis was performed to identify distinct DSHV-profiles, providing insight into which types of individuals may be more vulnerable to different forms of DSHV. The hierarchical clustering resulted in a 3-cluster solution for both, students and employees (see Fig. 1 and 2). Based on the mean values of each DSHV-category, clusters 1–3 were defined as follows: *I. No DSHV-experiences* (students: n = 650 [54%]; employees: n = 352 [54%]), *II. limited DSHV-experiences* (without sexual violence in employees) (students: n = 423 [35%]; employees: n = 222 [34%]), *III. extensive DSHV-experiences* with more sexual harassment and sexual violence (students: n = 124 [10%]; employees: n = 79 [12%]). The detailed DSHV-profiles of the three clusters can be found in the appendix.

### Validation of DSHV-profiles

The three clusters differed significantly (*p* < 0.001) in the frequency of each of the four forms of DSHV among students and employees. Individuals in the third cluster with extensive DSHV-experiences (for students and employees) were always the most frequently affected ones.

### Cluster affiliation

Among the students, there were significant differences between the three clusters depending on gender (*p* < 0.001; Cramér’s *V* = 0.29), migration in the family history (*p* = 0.037; Cramér’s *V* = 0.08), stage of study (*p* < 0.001; Cramér’s *V* = 0.10), field of study (*p* < 0.001; Cramér’s *V* = 0.14) and perceived hierarchy in the field of study (*p* < 0.001; *Χ*^*2*^ = 54.797). Among employees, there were significant differences in gender (*p* < 0.001; Cramér’s *V* = 0.25), duration of employment (*p* = 0.004; Cramér’s *V* = 0.17) and perceived hierarchy in the workplace (*p* < 0.001; *Χ*^*2*^ = 34.824) (see Tables 3 and 4).

**Table 3:** Differences between the three clusters (HCA) amongst students

	**Cluster I: no DSHV experiences**	**Cluster II: limited DSHV experiences**	**Cluster III: extensive DSHV experiences**	***p***	**Cramér`s* V*/*Chi***^***2***^
^13^Female gender (n = 1187)	49%	41%	91%	< 0.001**	.29
^1^Age (n = 1197)				0.169	-
18–24 years	69%	62%	63%		
25–29 years	24%	29%	29%		
30 years or older	7%	9%	10%		
^1^Migration in the family history (***n*** = 1179)	81%	78%	77%	0.037*	0.08
^1^Parents without university degree (***n*** = 821)	47%	48%	41%	0.577	-
^1^Study stage (***n*** = 1193)				< 0.001**	0.10
bachelor or similar	49%	36%	43%		
master or similiar	46%	55%	44%		
postgraduate	6%	9%	13%		
Field of study (***n*** = 1044)				< 0.001**	.14
natural sciences	39%	30%	35%		
psychology	14%	9%	8%		
economics	10%	4%	5%		
medical subjects	38%	57%	52%		
^2^Hierarchy at field of study (n = 1087)	*m* = 4.67 (*sd* = 2.07)	*m* = 5.54 (*sd* = 2.11)	*m* = 5.90 (*sd* = 2.09)	< 0.001**	54.797

^1^Chi square tests were calculated

^2^Kruskal-Wallis test was calculated

^3^Diverse gender was excluded because of small sample size

*indicates *p* < 0.05. **indicates *p* < 0.01

**Table 4:** Differences between the three clusters (HCA) amongst staff

	**Cluster I: no DSHV experiences**	**Cluster II: limited DSHV experiences**	**Cluster III: extensive DSHV experiences**	***p***	**Cramér`s* V*/*Chi***^***2***^
^13^Female gender (n = 650)	51%	66%	89%	< 0.001**	.25
^1^Age (n = 653)				0.411	-
18–35 years	40%	37%	46%		
36–50 years	29%	35%	25%		
> 50 years	31%	28%	29%		
^1^Migration in the family history (n = 638)	80%	75%	85%	0.153	-
^1^Parents without university degree (n = 635)	38%	41%	35%	0.582	-
^1^Leadership position (n = 628)	18%	22%	17%	0.407	-
^1^Duration of employment (n = 644)				0.004**	.17
< 1 year	14%	7%	1%		
1–5 years	35%	37%	32%		
6–10 years	16%	15%	15%		
> 10 years	35%	42%	52%		
^2^Hierarchy at workplace (n = 628)	*m* = 3.23 (*sd* = 2.20)	*m* = 4.08 (*sd* = 2.45)	*m* = 4.60 (*sd* = 2.46)	< 0.001**	34.824

^1^Chi square tests were calculated

^2^Kruskal–Wallis test was calculated

^3^Diverse gender was excluded because of small sample size

^**^indicates *p* < 0.01

The significant factors identified here were included in the two multinomial logistic regression models: among students, the factors female gender (*p* < 0.001), stage of study (*p* = 0.003), field of study (*p* = 0.004) and perceived hierarchy within their field of study (*p* < 0.001) significantly predicted cluster affiliation, highlighting how these factors interact to increase DSHV-risk. Among employees, female gender (*p* < 0.001), duration of employment (*p* = 0.002) and perceived hierarchy at the workplace (*p* < 0.001) significantly predicted cluster affiliation, highlighting the contribution of these factors to an increased DSHV-risk among employees. All of these significant factors added to the regression models – except family history of migration for students – significantly predicted cluster affiliation: students were more likely to be in the second cluster (limited DSHV-experiences) than in the first cluster (no DSHV-experiences; reference category) if they were female, if the perceived hierarchy in the field of study was more pronounced, if they were less likely to have studied for a bachelor's degree and if they had studied medical or natural subjects. Female gender was particularly significant for assignment to the third cluster (extensive DSHV-experiences) instead of the first cluster (no DSHV-experiences). Hierarchy, the postgraduate level and, again, studying a natural science subject were also significant (see Tables 5 and 6).

**Table 5:** Multinomial logistic regression, parameter estimates (students), *n* = 928, ** indicates *p* < 0.01

	**Wald**	**Sign**
Constant term	0.000	
Hierarchy at field of study	25.996	< 0.001**
Gender	57.116	< 0.001**
Migration	3.117	0.210
Study degree	15.892	0.003**
Field of study	18.572	0.005**

**Table 6:** Multinomial logistic regression, parameter estimates (students), *n* = 928, ** indicates *p* < 0.01, * indicates *p* < 0.05

**Cluster number**			**Wald**		**Sign.**	**Exp(B)**	**95% C.I. for Exp(B)**	
							Lower	Upper
2: limited DSHV experiences		**Constant term**	11.119		0.001**			
		**Hierarchy at field of study**	14.670		<0.001**	1.171	1.080	1.269
		**Gender**						
		Female	28.776		<0.001**	2.438	1.761	3.377
		Male	^b^0					
		**Migration**						
		No migration in the family history	2.965		0.085	0.731	0.512	1.044
		Migration in the family history	^**b**^0					
		**Study degree**						
		Study degree – Bachelor	7.633		0.006**	0.433	0.239	0.784
		Study degree – Master	2.701		0.100	0.612	0.341	1.099
		Study degree – Postgraduate	^b^0					
		**Field of study**						
		natural sciences	5.844		0.016*	1.886	1.128	3.156
		medical subjects	8.089		0.004**	2.061	1.252	3.393
		economics	0.303		0.582	0.801	0.363	1.766
		psychology	^b^0					
3: extensive DSHV experiences	**Constant term**		29.245	<0.001**				
	**Hierarchy at field of study**		17.554	<0.001**		1.339	1.168	1.534
	**Gender**							
	Female		29.543	<0.001**		7.151	3.518	14.536
	Male		^b^0					
	**Migration**							
	No migration in the family history		0.897	0.344		0.763	0.436	1.336
	Migration in the family history		^b^0					
	**Study degree**							
	Study degree – Bachelor		8.847	0.003**		0.293	0.131	0.658
	Study degree – Master		9.098	0.003**		0.289	0.129	0.648
	Study degree – Postgraduate		^b^0					
	**Field of study**							
	natural sciences		5.5057	0.025*		2.704	1.136	6.433
	medical subjects		2.163	0.141		1.884	0.810	4.380
	economics		0.720	0.396		1.700	0.499	5.797
	psychology		^b^0					

Among employees, women were more likely to be assigned to the second cluster (limited DSHV-experiences) rather than to the first (no DSHV-experiences; reference category), especially when they experienced a more pronounced hierarchy in the workplace and when they had been employed for more than ten years, compared to less than one year. These factors were particularly significant for assignment to the third cluster (extensive DSHV-experiences) rather than the first (no DSHV-experiences) (see Tables 7 and 8).

**Table 7:** Multinomial logistic regression, parameter estimates (staff), *n* = 618, ** indicates *p* < 0.01

	**Wald**	**Sign**
Constant term	0.000	
Hierarchy at field of work	24.397	< 0.001**
Gender	41.515	< 0.001**
Duration of employment	20.631	0.002**

**Table 8:** Multinomial logistic regression, parameter estimates (staff), *n* = 618, ** indicates *p* < 0.01, * indicates *p* < 0.05

**Cluster number**		**Wald**	**Sign**	**Exp(B)**	**95% C.I. for Exp(B)**	
					Lower	Upper
2: limited DSHV experiences	**Constant term**	24.594	< 0.001**			
	**Hierarchy at field of work**	13.129	< 0.001**	1.153	1.068	1.246
	**Gender**					
	Female	10.430	0.001**	1.830	1.268	2.640
	Male	^**b**^0				
	**Duration of employment**					
	Duration of employment: < 1 year	6.534	0.011*	0.405	0.202	0.810
	Duration of employment: 1–5 years	0.777	0.378	0.833	0.554	1.252
	Duration of employment: 6–10 years	2.637	0.104	0.610	0.373	1.097
	Duration of employment: > 10 years	^**b**^0				
3: extensive DSHV experiences	**Constant term**	60.758	< 0.001**			
	**Hierarchy at field of work**	19.001	< 0.001**	1.267	1.139	1.410
	**Gender**					
	Female	27.825	< 0.001**	7.346	3.501	15.411
	Male	^**b**^0				
	**Duration of employment**					
	Duration of employment: < 1 year	7.020	0.008**	0.064	0.008	0.488
	Duration of employment: 1–5 years	3.359	0.067	0.572	0.315	1.040
	Duration of employment: 6–10 years	3.555	0.059	0.479	0.223	1.030
	Duration of employment: > 10 years	^**b**^0				

^a^The reference cluster is 1 (no DSHV experiences)\

^b^This parameter is set to zero as it is redundant

## Discussion

This study provides the first comprehensive evidence of the co-occurrence of all forms of discrimination, stalking, sexual harassment and sexual violence (DSHV) at universities, including both students and employees. These findings underscore the structural and far-reaching implications of DSHV within academic environments. They provide a nuanced understanding of the interconnectedness of these experiences during academic careers. This reinforces previous findings that universities, as institutions pivotal in shaping regions, face high rates of sexual violence, discrimination and harassment [2, 4, 4, 5]. In particular, the operationalization of perceived hierarchy, for which there is a lack of research, proved to be highly relevant. Specific differences between the field of study were identified.

Notably, we observed high rates of DSHV-prevalence: among students, 40% reported experiences of discrimination, 13% of stalking, 33% of sexual harassment and 4% of sexual violence. Among employees, 46% reported experiences of discrimination,18% of stalking, 29% of sexual harassment and 6% of sexual violence. The prevalence rates for sexual harassment and sexual violence among students (29.3% and 3.5%) and employees (35.3% and 1.2%) were comparable in the UniSAFE survey [22]. In our sample, students were slightly more likely to experience sexual harassment than in the UniSAFE survey; employees were also more likely to experience sexual violence, but less likely to experience sexual harassment. The UniSAFE-data were collected more than a year in advance (in early 2022). Influences from the COVID-19 pandemic could possibly explain the differences, as data on the overall impact of the pandemic restrictions and gender-based violence on the population is available [45]. The prevalence rates for stalking were either lower than or comparable to those of a previous study on students [19], which also had a low response rate of 26%, with an average completion rate of only about 14% [19]. Consequently, the significance and comparability of the results are questionable. In both samples, higher rates of discrimination were found compared to the study by Führer and colleagues (2024). However, the invitation to their survey was unsystematic and a response rate could not be measured. It remains unclear how many people were even informed about the study. Furthermore, only n = 890 of the N = 1,671 were ultimately included in the analyses [18]. Therefore, the representativeness of these results is questionable.

Using a data-driven exploratory approach, we identified three distinct clusters of DSHV-experiences of similar forms among students and employees: an unaffected group, a group with limited DSHV-experiences and a group with extensive DSHV-experiences. Notably, all four forms of DSHV varied across the clusters, indicating the interconnected patterns of experiences of different forms of DSHV. Consequently, experiencing one form of DSHV may indicate further experiences of DSHV. Studies by Feltes and colleagues (2012) and Buhi, Clayton and the Heather Hepler (HH) Surrency MPH (2009) have already suggested that there may be a common occurrence. However, Feltes and colleagues (2012) did not include men, employees or experiences of discrimination. Buhi and colleagues (2009) only investigated rape and stalking. This point should be considered by colleagues, superiors, confidants and counselors when reporting a specific form of DSHV.

Furthermore, we identified the same significant risk factors for cluster affiliation in both samples - students and employees. In both groups, female gender, duration of time at the university and perceived workplace or study field hierarchy were significant. Female gender and duration of time at the university as significant factors for cluster affiliation confirm previous research (e.g. [5, 39]). As there is a lack of studies assessing perceived hierarchy, our findings support the hypothesis regarding the relevance of the hierarchy. This appears to be consistent with studies examining potentially related issues: Muhonen’s [46] research on gender harassment among university employees and fair leadership, operationalized by asking, for example, whether tasks are distributed fairly, showed that fair leadership was associated with less gender harassment for both men and women.

For students, the field of study emerged as an important factor. On the one hand, students in medical fields were particularly vulnerable to DSHV, consistent with previous findings that show higher prevalence rates of discrimination, stalking and sexual harassment among medical students [25, 26],National Academies of Sciences, Engineering and Medicine, 2018). On the other hand, our results also indicate that female students of natural sciences are at higher risk for DSHV-experiences, as they were associated with the most affected cluster. This extends previous findings demonstrating that medical students were more affected by sexual harassment than engineering students (National Academies of Sciences, Engineering and Medicine, 2018). In our sample, female students in the natural sciences were more likely to experience all forms of DSHV compared to their peers in medical fields. A lack of peer support, resulting from the high male-to-female ratio in these fields, may contribute to the higher rate of DSHV-experiences, as suggested by studies on gender-based violence in broader societal contexts [40]. Migration status did not show a significant effect for students when added to the complete model, although differences were observed between clusters. This may suggest that migration status is confounded by other, more relevant factors in determining cluster affiliation. Family educational background also did not significantly differ between the clusters, potentially due to complex interrelationships. Previous studies have identified both gender and age cohort effects in the relationship between family education level and individual educational attainment [38].

Implications.

Our findings underscore the need to evaluate the impact of structural changes, particularly those aimed at protecting female students and employees at universities from DSHV. We provide empirical support for calls to dismantle hierarchies [30, 31] and increase transparency in longer-lasting dependency relationships, such as during doctoral studies. A greater awareness for DSHV is needed with low-threshold access to complaints offices. The similar patterns of victimization among both students and employees further highlight the need for structural reforms. Notably, our results show that for both students and employees, experiencing one form of DSHV is associated with a higher likelihood of experiencing other forms, highlighting the interconnected nature of these issues. Additionally, subject-specific differences should be examined. Previous literature has already indicated that medical and, in some cases, natural science subjects are associated with a higher level of affectedness [18, 25–28, 29]. However, the finding that natural sciences are particularly affected is novel and likely a result of the integrative approach used in this study.

Efforts should focus on reducing hierarchies in both the workplace and in the field of study. Qualitative interviews with graduate students have highlighted similar issues after recording sexual violence and sexual harassment: The lack of trust in university processes highlights the need to dismantle hierarchies to achieve greater willingness to report experiences of sexual harassment or sexual violence [47]. Informing students and employees also appears to be important: there is evidence that students are often poorly informed about sexual violence and sexual harassment policies, especially at the beginning of their studies [48]. Faculty members are often the first point of contact for students experiencing mental health problems that may be related to DSHV. However, many report feeling unprepared or lacking the training needed to address these concerns effectively [49].

Strengths and limitations.

A major strength of this study is its comprehensive assessment of all four forms of DSHV among both students and employees, allowing for a detailed comparison between the two groups. However, due to the low participation rate among students, the results cannot be considered fully representative for the University of Ulm. Despite this, similarities in DSHV-patterns were observed between students and employees, who had a higher response rate. In addition, the data are based on self-reported information from students and employees. A mixed-methods approach might derive further information. Despite this, self-reporting remains a feasible method for capturing sensitive personal experiences on a large scale in academic settings, where privacy and ethical considerations are paramount and restrictions of this kind on data collection from related areas are not known to date [50]. Recall or recency bias cannot be ruled out, as the duration of employment or study at the university was not controlled for. We also inquired about incidents initiated by individuals affiliated with the University of Ulm. This may have resulted in incidents involving unknown perpetrators going unreported. Some of the events are not described using behaviorally-specific, explicit language. Despite the predominantly high level of education among the sample, it cannot be ruled out that questions were misunderstood or interpreted differently.

## Conclusion

DSHV occurs globally and in specific groups for students and employees: Risk factors for DSHV are stronger hierarchies and time spent at the university, as well as specific fields of study, such as medical subjects and natural sciences, and female gender. One form of DSHV-experience may be associated with other and different forms of DSHV. Similar patterns of DSHV-experiences can be seen in students and employees. Therefore, measures against DSHV should incorporate both global and risk group-related, targeted approaches based on a risk analysis and might be especially helpful for female students and employees.

## Supplementary Information

Supplementary Material 1.

Supplementary Material 2.

## Data Availability

The datasets used and/or analysed during the current study are available from the corresponding author on reasonable request.

## References

[1] Chatterton P. The cultural role of universities in the community: revisiting the university—community debate. Environ Plann A. 2000;32(1):165–81. 10.1068/a3243.

[2] Doyle L. The role of universities in the ‘Cultural Health’ of their regions: universities’ and regions’ understandings of cultural engagement. Eur J Educ. 2010;45(3):466–80. 10.1111/j.1465-3435.2010.01441.x.

[3] Gunasekara C. The third role of Australian universities in human capital formation. J High Educ Policy Manag. 2004;26(3):329–43. 10.1080/1360080042000290186.

[4] Boliver V, Powell M. Fair admission to universities in England: Improving policy and practice. Nuffield Foundation. 2021; https://www.nuffieldfoundation.org/wp-content/uploads/2021/01/Fair-admission-to-universities-in-England.pdf.

[5] Wood L, Hoefer S, Kammer-Kerwick M, Parra-Cardona JR, Busch-Armendariz N. Sexual harassment at institutions of higher education: prevalence, risk, and extent. J Interpers Violence. 2021;36(9–10):4520–44. 10.1177/0886260518791228. 30071790 10.1177/0886260518791228 PMC10676016

[6] Frank E, Brogan D, Schiffman M. Prevalence and correlates of harassment among US women physicians. Arch Intern Med. 1998;158(4):352–8. 10.1001/archinte.158.4.352. 9487232 10.1001/archinte.158.4.352

[7] Klein L B, Martin S L. Sexual harassment of college and university students: A systematic review. Trauma, Violence, & Abuse. 2021; 22(4), 777–792, https://doi.org/10.1177/1524838019881731.36. Koss, M. P., Swartout, K. M., Lopez, E. C., Lamade, R. V., Anderson, E. J., Brennan, C. L., & Prentky, R. A. (2022). The scope of rape victimization and perpetration among national samples of college students across 30 years. *Journal of interpersonal violence*, *37*(1–2), NP25- NP47. 10.1177/08862605211050103. 10.1177/08862605211050103 34911373

[8] Lipinsky A, Schredl C, Baumann H, Humbert A, Tanwar J. Gender-based violence and its consequences in European Academia, Summary results from the UniSAFE survey. 2022. Report, November 2022. UniSAFE project no.101006261.

[9] Posselt J. Discrimination, competitiveness, and support in US graduate student mental health. Stud Grad Postdoc Educ. 2021;12(1):89–112.

[10] Sheldon E, Simmonds-Buckley M, Bone C, Mascarenhas T, Chan N, Wincott M, et al. Prevalence and risk factors for mental health problems in university undergraduate students: a systematic review with meta-analysis. J Affect Disord. 2021;287:282–92. 10.1016/j.jad.2021.03.054. 33812241 10.1016/j.jad.2021.03.054

[11] Henning MA, Zhou C, Adams P, Moir F, Hobson J, Hallett C, et al. Workplace harassment among staff in higher education: a systematic review. Asia Pac Educ Rev. 2017;18:521–39. 10.1007/s12564-017-9499-0.

[12] Cortina LM, Swan S, Fitzgerald LF, Waldo C. Sexual harassment and assault: chilling the climate for women in academia. Psychol Women Q. 1998;22(3):419–41.

[13] Banyard VL, Demers JM, Cohn ES, Edwards KM, Moynihan MM, Walsh WA, et al. Academic correlates of unwanted sexual contact, intercourse, stalking, and intimate partner violence: An understudied but important consequence for college students. J Interpers Violence. 2022;35(21–22):4375–92. 10.1177/0886260517715022. 10.1177/0886260517715022 29294800

[14] Morgan RK. Students stalking faculty: real and imagined relationships. Sex Cult. 2010;14:5–16.

[15] Morgan RK, Kavanaugh KD. Student stalking of faculty: results of a nationwide survey. Coll Stud J. 2011;45(3):512–24.

[16] Buhi ER, Clayton H, Surrency HH. Stalking victimization among college women and subsequent help-seeking behaviors. J Am Coll Health. 2009;57(4):419–26. 10.3200/JACH.57.4.419-426. 19114381 10.3200/JACH.57.4.419-426

[17] Stevens C, Liu CH, Chen JA. Racial/ethnic disparities in US college students’ experience: discrimination as an impediment to academic performance. J Am Coll Health. 2018;66(7):665–73. 10.1080/07448481.2018.1452745. 29565755 10.1080/07448481.2018.1452745

[18] Führer A, Wagner K, Reinhardt Z, Wienke A. Under the radar: a survey of students’ experiences of discrimination in the German University context. Educ Sci. 2024;14(6):602. 10.3390/educsci14060602.

[19] Fedina L, Backes BL, Sulley C, Wood L, Busch-Armendariz N. Prevalence and sociodemographic factors associated with stalking victimization among college students. J Am Coll Health. 2020;68(6):624–30. 10.1080/07448481.2019.1583664. 30908169 10.1080/07448481.2019.1583664

[20] Jordan CE, Wilcox P, Pritchard AJ. Stalking acknowledgement and reporting among college women experiencing intrusive behaviors: implications for the emergence of a “classic stalking case.” J Crim Justice. 2007;35(5):556–69. 10.1016/j.jcrimjus.2007.07.008.

[21] Feltes, List, Schneider, Höfker. "Gender-based Violence, Stalking and Fear of Crime.", in: Länderbericht Deutschland. 2012. (Bochum).

[22] Humbert A L, Ovesen N, Simonsson A, Strid S, Hearn J, Huck A, Andreska Z, Linková M, Pilinkaitė Sotirovič V, Blažytė G, Pereira B. UniSAFE D6.1: Report on the multi-level analysis and integrated dataset. Zenodo. 2022. 10.5281/zenodo.7540229.

[23] Agardh A, Priebe G, Emmelin M, Palmieri J, Andersson U, Östergren PO. Sexual harassment among employees and students at a large Swedish university: who are exposed, to what, by whom and where–a cross-sectional prevalence study. BMC Public Health. 2022;22(1):2240. 36456935 10.1186/s12889-022-14502-0 PMC9714219

[24] Coulter RW, Mair C, Miller E, Blosnich JR, Matthews DD, McCauley HL. Prevalence of past-year sexual assault victimization among undergraduate students: exploring differences by and intersections of gender identity, sexual identity, and race/ethnicity. Prev Sci. 2017;18:726–36. 10.1007/s11121-017-0762-8. 28210919 10.1007/s11121-017-0762-8 PMC5511765

[25] Carr PL, Ash AS, Friedman RH, Szalacha L, Barnett RC, Palepu A, et al. Faculty perceptions of gender discrimination and sexual harassment in academic medicine. Ann Intern Med. 2000;132(11):889–96. 10836916 10.7326/0003-4819-132-11-200006060-00007

[26] Fnais N, Soobiah C, Chen MH, Lillie E, Perrier L, Tashkhandi M, et al. Harassment and discrimination in medical training: a systematic review and meta-analysis. Acad Med. 2014;89(5):817–27. 24667512 10.1097/ACM.0000000000000200

[27] Jendretzky K, Boll L, Steffens S, Paulmann V. Medical students’ experiences with sexual discrimination and perceptions of equal opportunity: a pilot study in Germany. BMC Med Educ. 2020;20(1):1–12. 10.1186/s12909-020-1952-9. 10.1186/s12909-020-1952-9 PMC7036258 32087726

[28] Schoenefeld E, Marschall B, Paul B, Ahrens H, Sensmeier J, Coles J, et al. Medical education too: sexual harassment within the educational context of medicine–insights of undergraduates. BMC Med Educ. 2021;21(1):1–6. 10.1186/s12909-021-02497-y. 33526025 10.1186/s12909-021-02497-y PMC7852293

[29] National Academies of Sciences, Engineering, and Medicine. Sexual harassment of women: Climate, culture, and consequences in academic sciences, engineering, and medicine. Washington, DC: National Academies Press, 2018. Available at: 10.17226/24994 Accessed September 30, 2024. 29894119

[30] Benya F F, Widnall S E, Johnson P A (Eds.). Sexual harassment of women: Climate, culture, and consequences in academic sciences, engineering, and medicine, 2018. 29894119

[31] Berdahl JL. Harassment based on sex: protecting social status in the context of gender hierarchy. Acad Manage Rev. 2007;32(2):641–58. 10.5465/amr.2007.24351879.

[32] Kabat-Farr D, Cortina LM. Sex-based harassment in employment: new insights into gender and context. Law Hum Behav. 2014;38(1):58–72. 10.1037/lhb0000045. 23914922 10.1037/lhb0000045

[33] Poyrazli S, Lopez MD. An exploratory study of perceived discrimination and homesickness: a comparison of international students and American students. J Psychol. 2007;141(3):263–80. 10.3200/JRLP.141.3.263-280. 17564257 10.3200/JRLP.141.3.263-280

[34] Kearney LK, Gilbert LA. The role of ethnicity in Mexican American and non-Hispanic White students’ experience of sexual harassment. Hispanic J Behav Sci. 2012;34(4):507–24. 10.1177/0739986312461134.

[35] Buchanan NT, Bergman ME, Bruce TA, Woods KC, Lichty LL. Unique and joint effects of sexual and racial harassment on college students’ well-being. Basic Appl Soc Psychol. 2009;31(3):267–85. 10.1080/01973530903058532.

[36] Yoon E, Stiller Funk R, Kropf NP. Sexual harassment experiences and their psychological correlates among a diverse sample of college women. Affilia. 2010;25(1):8–18. 10.1177/0886109909354979.

[37] Gobena GA. Family socio-economic status effect on students’ academic achievement at college of education and behavioral sciences, Haramaya University, eastern Ethiopia. Journal of Teacher Education and Educators. 2018;7(3):207–22.

[38] Buchmann C, DiPrete TA. The growing female advantage in college completion: the role of family background and academic achievement. Am Sociol Rev. 2006;71(4):515–41. 10.1177/000312240607100401.

[39] Kammer-Kerwick M, Wang A, McClain TS, Hoefer S, Swartout KM, Backes B, et al. Sexual violence among gender and sexual minority college students: the risk and extent of victimization and related health and educational outcomes. J Interpers Violence. 2021;36(21–22):10499–526. 10.1177/0886260519883866. 31686584 10.1177/0886260519883866

[40] Heise L, Ellsberg M, Gottmoeller M. A global overview of gender-based violence. Int J Gynaecol Obstet. 2002;78:S5-14. 10.1016/S0020-7292(02)00038-3. 12429433 10.1016/S0020-7292(02)00038-3

[41] Kaladelfos A, Featherstone L. Sexual and gender-based violence: definitions, contexts, meanings. Aust Fem Stud. 2014;29(81):233–7. 10.1080/08164649.2014.958121.

[42] Lipinsky A, Schredl C, Baumann H, Lomazzi V, Freund F, Humbert A L, Tanwar J, Bondestam F, Kreßner F, Perez M. UniSAFE-Survey Questionnaire. Language Version: German. 2023, https://search.gesis.org/research_data/SDN-10.7802–2475?doi=10.7802/2475.

[43] Cohen J. Statistical power analysis for the behavioral sciences (2nd ed.). 1988. Hillsdale, N.J.: L. Erlbaum Associates, 10.4324/9780203771587.

[44] Ellis PD. The essential guide to effect sizes: Statistical power, meta-analysis, and the interpretation of research results. Cambridge university press; 2010. 10.1017/CBO9780511761676.

[45] Rodriguez-Jimenez R, Fares-Otero NE, García-Fernández L. Gender-based violence during COVID-19 outbreak in Spain. Psychol Med. 2023;53(1):299–300. 10.1017/S0033291720005024. 33280627 10.1017/S0033291720005024 PMC7804072

[46] Muhonen T. Exploring gender harassment among university teachers and researchers. J Appl Res High Educ. 2016;8(1):131–42. 10.1108/JARHE-04-2015-0026.

[47] Bloom BE, Sorin CR, Wagman JA, Oaks L. Employees, advisees, and emerging scholars: a qualitative analysis of graduate students’ roles and experiences of sexual violence and sexual harassment on college campuses. Sexuality & Culture. 2021;25:1653–72. 10.1007/s12119-021-09841-w. 34776727 10.1007/s12119-021-09841-w PMC8550674

[48] Bloom BE, Sorin CR, Oaks L, Wagman JA. Graduate students’ knowledge and utilization of campus sexual violence and sexual harassment resources. J Am Coll Health. 2023;71(5):1328–31. 10.1080/07448481.2021.1942010. 34242553 10.1080/07448481.2021.1942010

[49] Gulliver A, Farrer L, Bennett K, Ali K, Hellsing A, Katruss N, et al. University staff experiences of students with mental health problems and their perceptions of staff training needs. J Ment Health. 2018;27(3):247–56. 10.1080/09638237.2018.1466042. 29722579 10.1080/09638237.2018.1466042

[50] Tanton C, Bhatia A, Pearlman J, Devries K. Increasing disclosure of school-related gender-based violence: lessons from a systematic review of data collection methods and existing survey research. BMC Public Health. 2023;23(1):1012. 10.1186/s12889-023-15526-w. 37254071 10.1186/s12889-023-15526-w PMC10227976

