# Supplementary material for: Discrimination, stalking, sexual harassment and sexual violence at the university – exploring and predicting pattern-based subcategories among students and staff in a German university sample
Source: BMC Public Health. 2025 Dec 8;26:117. doi: 10.1186/s12889-025-25864-6 (PMC12781331; doi:10.1186/s12889-025-25864-6)
Supplement: Supplementary file 1 — Supplementary Material 1. [file 12889_2025_25864_MOESM1_ESM.docx]

Attachment Questionnaire (sections on experiences of discrimination and stalking)

*Headline/Trigger Warning: The next set of questions addresses your personal experience with different forms of discrimination, harassment and violence. You may not perceive all situations we ask about as discrimination, harassment and violence, however, your experiences with these incidents are important for this survey. Some of the wording used in this survey is explicit and some people may find it uncomfortable or distressing. It is important that we ask the questions in this way so that it is clear what we mean. You have the option to skip questions if you do not feel comfortable answering them. Information on how to get help, if you need it, appears at the end of the survey or if you click here.*

We would now like to ask you some questions about your personal experiences of discrimination by persons **associated with Ulm University**.

Have you ever experienced discrimination by a person/persons associated with Ulm University based on the following characteristics: (*Please choose at least one answer for each line):*

|  | *Yes, in the last 12 months* | *Yes, at some point during your time at Ulm University* | *No* |
| --- | --- | --- | --- |
| Social origin |  |  |  |
| Skin colour |  |  |  |
| National or ethnic origin |  |  |  |
| Gender |  |  |  |
| Sexual orientation |  |  |  |
| Sexual identity |  |  |  |
| Religion |  |  |  |
| Political views |  |  |  |
| Physical or mental illness |  |  |  |
| Disability |  |  |  |
| Family planning/parental leave |  |  |  |
| Age |  |  |  |
| Discrimination for other reasons (FREITEXTFELD) [Please avoid information that could identify you or others]. |  |  |  |
| Prefer not to say | | | |

We would now like to ask you some questions about your personal experiences with stalking by persons **associated with Ulm University**. You have the option to skip any questions if you do not feel comfortable answering them. Information on how to get help, if you need it, appears at the end of the survey or if you click here.

Since you started at Ulm University, has someone associated with Ulm University ever done any of the following to you? *(Please choose at least one answer for each line.)*

|  | *Yes, in the last 12 months* | *Yes, at some point during your time at Ulm University* | *No* |
| --- | --- | --- | --- |
| Unwanted, disproportionately frequent contact or 'waylaying'? |  |  |  |
| Surveillance, suspicious watching or pursuit |  |  |  |
| Unwanted or inappropriate phone calls, emails, letters, notes |  |  |  |
| Unwanted gifts |  |  |  |
| Unwanted preferential treatment, favours, attention |  |  |  |
| Other (FREITEXTFELD) [Please avoid information that could identify you or others]. |  |  |  |
| Prefer not to say | | | |

If you have had multiple experiences with stalking by a person/persons associated with Ulm University, please relate the following questions to the experience that happened most recently.
