# Supplementary material for: Discrimination, stalking, sexual harassment and sexual violence at the university – exploring and predicting pattern-based subcategories among students and staff in a German university sample
Source: BMC Public Health. 2025 Dec 8;26:117. doi: 10.1186/s12889-025-25864-6 (PMC12781331; doi:10.1186/s12889-025-25864-6)
Supplement: Supplementary file 2 — Supplementary Material 2. [file 12889_2025_25864_MOESM2_ESM.docx]

|  | ^1^Analysis sample | ²Response rate in the respective category of the total student body |
| --- | --- | --- |
|  | N=1358 | 14 % |
| Age group |  |  |
| 18-24 | 897 (66 %) | 17 % |
| 25-29 | 356 (26 %) | 12 % |
| 30 or older | 105 (8 %) | 8 % |
| Gender |  |  |
| female | 816 (60 %) | 17 % |
| Male | 530 (39 %) | 12 % |
| diverse/other | 12 (1 %) | Official numbers <12 |
| Study stage |  |  |
| Bachelor | 590 (43 %) | 15 % |
| Master | 657 (48 %) | 17 % |
| Postgradual | 106 (8 %) | 7 % |
| No information | 5 (<1%) |  |
| ^3^Study subject (multiple entries possible) |  |  |
| Biology | 138 | 16 % |
| Chemistry | 110 | 17 % |
| Informatics | 161 | 16 % |
| Engineering | 49 | 13 % |
| Mathematics | 118 | 19 % |
| Medicine | 465 | 16 % |
| Dental medicine | 29 | 7 % |
| Other medical subjects | 58 | 13 % |
| Physics | 54 | 17 % |
| Psychology | 132 | 15 % |
| Economics | 90 | 11 % |

Table S1. Composition of the student analysis sample (N=1358). ^1^Percentage in brackets corresponds to the proportion of the analysis sample (N=1358). ^2^Percentage corresponds to the proportion of the respective category of the total sample. ^3^The total number (N=1404) is greater than the number of participants due to the option of multiple responses. The relative proportion in the analysis sample is not calculated. Teacher training students were asked to tick all applicable subjects. ^4^In addition to teacher training students, for whom there was no separate selection option, students who are currently in an orientation semester or taking a language course, for example, and who were not assigned to a department at the time of the survey were also contacted. Their total number, together with the teacher training students, amounts to n=183. It is unclear how many of these students participated in the survey.

|  | **Number of experiences with forms of discrimination (n = 1197)** | | | | | | | | | | | | | | | |  |
| --- | --- | --- | --- | --- | --- | --- | --- | --- | --- | --- | --- | --- | --- | --- | --- | --- | --- |
|  | social origin | skin colour | nationality | | gender | | sexual orientation | sexual  identity | religion | | politics | disease | disability | family  planning | age | | others |
| Cluster I: no DSHV experiences (n = 650) | 0% | 0% | 0% | | 0% | | 0% | 0% | 0% | | 0% | 0% | 0% | 0% | 0% | | 0% |
| Cluster II: limited DSHV experiences (n = 423) | 10% | 4% | 13% | | 35% | | 4% | 4% | 6% | | 11% | 12% | 2% | 7% | 14% | | 8% |
| Cluster III: extensive DSHV experiences (n = 124) | 18% | 7% | 17% | | 65% | | 21% | 11% | 14% | | 21% | 25% | 2% | 9% | 21% | | 7% |
|  | **Number of experiences with forms of stalking (n = 1197)** | | | | | | | | | | | | | | | |  |
|  | unwilled contact or ‚lurking‘ | surveillance, observation or persecution | unwilled phone calls, e-mails, letters, notes | | | unwilled gifts | unwilled preferential treatment | others |  | |  |  |  |  | |  |  |
| Cluster I: no DSHV experiences (n = 650) | 0% | 0% | 0% | | | 0% | 0% | 0% |  | |  |  |  |  | |  |  |
| Cluster II: limited DSHV experiences (n = 423) | 6% | 3% | 8% | | | 1% | 4% | 1% |  | |  |  |  |  | |  |  |
| Cluster III: extensive DSHV experiences (n = 124) | 27% | 14% | 21% | | | 9% | 21% | 5% |  | |  |  |  |  | |  |  |
|  | **Number of experiences with forms of sexual harrassment (n = 1197)** | | | | | | | | | | | | | | | |  |
|  | inappropriate private questions | stared / inappropriate contacted | sexually comments / jokes | intrusive comments about appearance | | | inappropriate invitations | exposed him- / herself to you | unwilled seen pornographic material | | unwilled touched, hugged, kissed | others |  |  | |  |  |
| Cluster I: no DSHV experiences (n = 650) | 0% | 0% | 0% | 0% | | | 0% | 0% | 0% | | 0% | 0% |  |  | |  |  |
| Cluster II: limited DSHV experiences (n = 423) | 14% | 13% | 24% | 7% | | | 4% | 1% | 1% | | 9% | 1% |  |  | |  |  |
| Cluster III: extensive DSHV experiences (n = 124) | 69% | 89% | 86% | 76% | | | 36% | 3% | 8% | | 48% | 8% |  |  | |  |  |
|  | **Number of experiences with forms of sexual violence (n = 1197)** | | | | | | | | | | | | | | | |  |
|  | tried to extort sexual favors in return | extorted sexual favors in return | tried to force sexual intercourse | | forced sexual intercourse | | forced to any form of sexual activity | forced to any form of sexual activity without ability to consent | | others |  |  |  |  | |  |  |
| Cluster I: no DSHV experiences (n = 650) | 0% | 0% | 0% | | 0% | | 0% | 0% | | 0% |  |  |  |  | |  |  |
| Cluster II: limited DSHV experiences (n = 423) | <1% | <1% | <1% | | 1% | | 1% | 1% | | 1% |  |  |  |  | |  |  |
| Cluster III: extensive DSHV experiences (n = 124) | 2% | 1% | 5% | | 2% | | 7% | 9% | | 3% |  |  |  |  | |  |  |

Table S2. Frequencies of experiences of different forms of discrimination, stalking, sexual harassment and violence amongst students according to the three cluster solution (HCA).

|  | **Number of experiences with forms of discrimination (n = 625)** | | | | | | | | | | | | | | | |  |
| --- | --- | --- | --- | --- | --- | --- | --- | --- | --- | --- | --- | --- | --- | --- | --- | --- | --- |
|  | social origin | skin colour | nationality | | gender | | sexual orientation | sexual  identity | religion | | politics | disease | disability | family  planning | age | | others |
| Cluster I: no DSHV experiences (n = 352) | 0% | 0% | 0% | | 0% | | 0% | 0% | 0% | | 0% | 0% | 0% | 0% | 0% | | 0% |
| Cluster II: limited DSHV experiences (n = 222) | 7% | 4% | 15% | | 41% | | 3% | 3% | 4% | | 10% | 11% | 5% | 21% | 14% | | 14% |
| Cluster III: extensive DSHV experiences (n = 79) | 17% | 10% | 13% | | 53% | | 11% | 5% | 14% | | 14% | 17% | 9% | 19% | 22% | | 5% |
|  | **Number of experiences with forms of stalking (n = 625)** | | | | | | | | | | | | | | | |  |
|  | unwilled contact or ‚lurking‘ | surveillance, observation or persecution | unwilled phone calls, e-mails, letters, notes | | | unwilled gifts | unwilled preferential treatment | others |  | |  |  |  |  | |  |  |
| Cluster I: no DSHV experiences (n = 352) | 0% | 0% | 0% | | | 0% | 0% | 0% |  | |  |  |  |  | |  |  |
| Cluster II: limited DSHV experiences (n = 222) | 9% | 6% | 10% | | | 4% | 2% | 1% |  | |  |  |  |  | |  |  |
| Cluster III: extensive DSHV experiences (n = 79) | 20% | 4% | 25% | | | 18% | 17% | 3% |  | |  |  |  |  | |  |  |
|  | **Number of experiences with forms of sexual harrassment (n = 625)** | | | | | | | | | | | | | | | |  |
|  | inappropriate private questions | stared / inappropriate contacted | sexually comments / jokes | intrusive comments about appearance | | | inappropriate invitations | exposed him- / herself to you | unwilled seen pornographic material | | unwilled touched, hugged, kissed | others |  |  | |  |  |
| Cluster I: no DSHV experiences (n = 352) | 0% | 0% | 0% | 0% | | | 0% | 0% | 0% | | 0% | 0% |  |  | |  |  |
| Cluster II: limited DSHV experiences (n = 222) | 8% | 2% | 14% | 4% | | | 1% | 0% | 1% | | 2% | 0% |  |  | |  |  |
| Cluster III: extensive DSHV experiences (n = 79) | 62% | 54% | 81% | 56% | | | 27% | 3% | 9% | | 28% | 10% |  |  | |  |  |
|  | **Number of experiences with forms of sexual violence (n = 625)** | | | | | | | | | | | | | | | |  |
|  | tried to extort sexual favors in return | extorted sexual favors in return | tried to force sexual intercourse | | forced sexual intercourse | | forced to any form of sexual activity | forced to any form of sexual activity without ability to consent | | others |  |  |  |  | |  |  |
| Cluster I: no DSHV experiences (n = 352) | 0% | 0% | 0% | | 0% | | 0% | 0% | | 0% |  |  |  |  | |  |  |
| Cluster II: limited DSHV experiences (n = 222) | 0% | 0% | 0% | | 0% | | 0% | 0% | | 0% |  |  |  |  | |  |  |
| Cluster III: extensive DSHV experiences (n = 79) | 1% | 0% | 3% | | 0% | | 4% | 1% | | 4% |  |  |  |  | |  |  |

Table S3. Frequencies of experiences of different forms of discrimination, stalking, sexual harassment and violence amongst staff according to the three cluster solution (HCA).
